# Diagnosis and Management of Infections in Patients with Mendelian Susceptibility to Mycobacterial Disease

**DOI:** 10.3390/pathogens13030203

**Published:** 2024-02-25

**Authors:** Aparna Dalvi, Umair Ahmed Bargir, Gita Natraj, Ira Shah, Manisha Madkaikar

**Affiliations:** 1 Department of Pediatric Immunology, ICMR National Institute of Immunohaematology, Mumbai 400012, India; aparna.dalvi@yahoo.co.in (A.D.); bargir.ua@icmr.gov.in (U.A.B.); 2 Seth GS Medical College and KEM Hospital, Mumbai 400012, India; gitanataraj@gmail.com; 3 Bai Jerbai Wadia Hospital for Children, Mumbai 400012, India; irashah@pediatriconcall.com

**Keywords:** mendelian susceptibility to mycobacterial disease, non-tuberculous mycobacteria, mycobacterium tuberculosis complex, Bacillus Calmette-Guérin vaccine

## Abstract

The diagnosis and treatment of patients with mendelian susceptibility to mycobacterial disease (MSMD) pose consistent challenges due to the diverse infection spectrum observed in this population. Common clinical manifestations include Bacillus Calmette-Guérin vaccine (BCG) complications in countries where routine BCG vaccination is practiced, while in non-BCG-vaccinating countries, Non-Tuberculous Mycobacteria (NTM) is prevalent. In tuberculosis-endemic regions, Mycobacterium tuberculosis (MTB) has a high prevalence, along with other intracellular organisms. Isolating these organisms presents a significant challenge, and treatment is often initiated without confirming the specific species. This review primarily focuses on the methods and challenges associated with diagnosing and treating MSMD patients.

## 1. Introduction

Inborn errors of immunity (IEI) are a group of disorders that result in an impaired immune response [1]. IEIs are classified by the International Union of Immunology Societies (IUIS) depending on the clinical and immunological evaluation of the defects. Mendelian susceptibility to mycobacterial diseases (MSMD) is a rare IEI that is grouped under defects of intrinsic and innate immunity [2,3]. MSMD predisposes to clinical disease caused by weakly virulent mycobacteria such as the Bacillus Calmette-Guérin (BCG) vaccine, nontuberculous mycobacteria (NTM), or environmental mycobacteria (EM). Infections due to more virulent *M. tuberculosis* have also been reported in MSMD patients. These patients present early in childhood, whereas milder forms can have an onset in adulthood and even remain asymptomatic. Mycobacterial diseases range from localized to disseminated or persistent life-threatening infections. Patients with MSMD may also suffer from infections due to intramacrophagic bacteria, fungi, parasites, and viruses [4,5,6,7,8,9,10,11]. Mutations in 21 different genes (*IFNGR1*, *IFNGR2*, *IFNG*, *IL12RB1*, *IL12RB2*, *IL23R*, *IL12B*, *ISG15*, *USP18*, *ZNFX1*, *TBX21*, *STAT1*, *TYK2*, *IRF8*, *CYBB*, *JAK1*, *RORC*, *NEMO*, *SPPL2A*, *MCTS1*, and *IRF1*) with more than 35 different genetic etiologies of MSMD have been described [12,13,14,15,16,17,18]. These deficiencies exhibit either autosomal recessive, autosomal dominant, or X-linked patterns of inheritance. Confirming the underlying genetic defects in these patients provides a better therapeutic approach and genetic counseling for the affected families. In most suspected MSMD cases, patients are treated empirically without confirming a microbiological and molecular diagnosis. This review mainly focuses on approaches and challenges associated with diagnosing and managing MSMD patients.

## 2. Pathophysiology and Clinical Manifestations of MSMD

**Figure 1:**
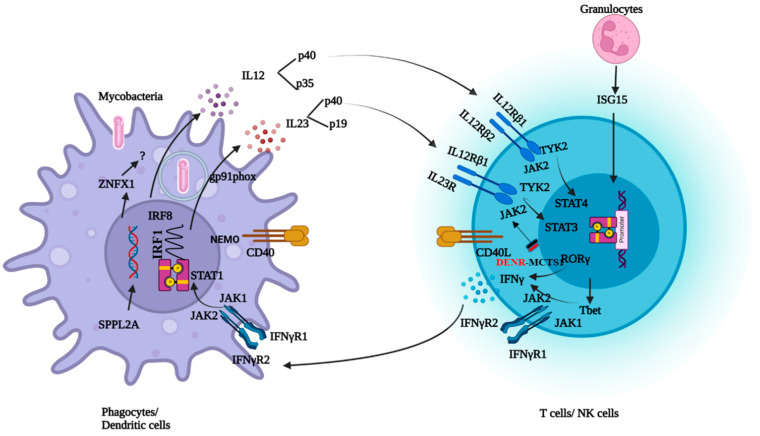
Schematic representation of genes involved in IFNγ response and production.

Type I cytokines such as IFNγ, IL12, and IL23 play a crucial role in the control of intracellular microorganisms. Once the bacteria are internalized, macrophages recognize the bacterial pathogen-associated molecular patterns and become activated. This activation produces cytokines such as IL12, IL23, and tumor necrosis factor-α (TNF-α). TNF-α plays a role in the formation of granulomas. IL12 leads to the differentiation of T cells into Th1 subpopulations and induces IFNγ secretion. IFNγ further activates the macrophages to eliminate the bacteria. In 1996, the discovery of IFNγR1 deficiency was reported as the first genetic etiology of MSMD with BCG infection. Over the period of discoveries of new genes in MSMD, this condition has been termed an inborn error of IFNγ immunity [7]. To mount an effective immune response against mycobacterial infections, the integrity of the IL12/23/ISG15-IFN-γ circuit is necessary (Figure 1). Mutations in the 21 genes involved in this circuit lead to either an impaired response or the production of IFNγ [8]. Molecular defects in the *IL12RB1* and *IFNGR1* genes together constitute about 80% of MSMD cases [9].

IL12Rβ1 deficiency is the most common genetic cause of MSMD. IL12 and IL-23 share a common IL12Rβ1 chain encoded by *IL12RB1*. Therefore, IL12 and IL23 signaling are defective in patients with IL12Rβ1 deficiencies. The clinical phenotype ranges from early deaths in infancy to asymptomatic adulthood, suggesting an incomplete penetrance [19,20]. Patients are vulnerable to mycobacterial disease, *Salmonella* infections, and chronic mucocutaneous candidiasis (CMC). About 25% of the patients with IL12Rβ1 deficiency develop fungal infections, including CMC [8,9,10]. It is unclear why only some patients with MSMD show susceptibility to fungal infections. Patients with IL-12p40, IL-12Rβ1, or IL-23R deficiency exhibit normal levels of circulating leukocytes from various subsets, including cell types that typically express IL-23R, such as NK, iNKT, mucosal-associated invariant T (MAIT), Vδ2+ γδ T, TH1, TH17, and TH1* cells. However, the susceptibility of certain individuals with deficiencies in *IL-12B*, *IL-12Rβ1*, or *IL-23R* to chronic mucocutaneous candidiasis (CMC) indicates the crucial role of human IL-23 in defending against mucocutaneous *Candida* spp. infections mediated by IL-17A/F. Recent findings showed that IL-23 induces IL-17A exclusively in MAIT cells from healthy individuals, a response absent in IL-23R-deficient patients. The incomplete penetrance of CMC in these patients is not solely explained by individual variability in IL-17A/F production by leukocyte subsets. Instead, it may be due to the preservation of C. albicans-specific memory CD4+ T cell development in IL-23R-deficient patients and the fact that IL-17A induction is governed by IL-23 in only one lymphocyte subset (MAIT cells), at least in ex vivo conditions [21].

In IL12Rβ1 patients, other clinical manifestations, such as autoimmunity and inflammatory bowel disease (IBD), have been described. Impairment in IL12Rβ1 signaling led to deficiencies in the differentiation of Th17 cells, which are vital for maintaining intestinal homeostasis. Th17 cells contribute to this balance by producing key cytokines, including IL-6, TGF-β, IL-17, and IL-1β, particularly in mucosal damage scenarios. They also play a pivotal role in defending against extracellular pathogens and stimulating the release of antimicrobial peptides from intestinal epithelial cells. The observed reduction in the Th17 cell population in the patient strongly suggests its potential involvement in the development of inflammatory bowel disease in this individual, aligning with the clinical manifestations seen in IL-12Rb1 patients, such as autoimmunity and IBD [22,23,24].

The second most common cause of MSMD is IFNγR1 deficiency. Two forms of autosomal recessive complete IFNγR1 deficiencies are described, with the presence of a non-functional receptor or a receptor that does not express at the cell surface. These complete forms of IFNγR1 are severe forms of MSMD. Patients present early, before 3 years of age, and suffer from disseminated BCG and/or EM with poorly characterized granuloma lesions. Infections with M. tuberculosis, viruses, and bacteria have also been reported [25,26,27,28]. In partial IFNγR1 deficiencies, the receptor is expressed on the cell surface, but the response to IFNγ is impaired. These are less severe forms than a complete IFNγR1 deficiency. In AD with partial IFNγR1 deficiency, there is an accumulation of truncated receptors on the surface due to a mutation in the intracellular domain lacking a receptor recycling motif. Higher amounts of protein are detected on the surface. Multifocal osteomyelitis is commonly seen in these patients. Recently, it was reported that AD IFNγR1-deficient and AD STAT1-deficient cells exhibit resistance to the inhibitory effects of IFNγ on osteoclast differentiation and bone resorption. Additionally, the IFNγ induced inhibition of NFATC1 mRNA, a critical transcription factor involved in osteoclast differentiation, was compromised in both AD IFNγR1-deficient and AD STAT1-deficient cells. These findings suggest that the increased osteoclastogenesis is responsible for the occurrence of osteomyelitis in these patients. Osteomyelitis is relatively rare in patients with complete IFNγR1 and STAT1 deficiency. Both complete AR IFNγR1 deficiency and complete AR STAT1 deficiency are life-threatening conditions, necessitating early intervention through hematologic stem cell transplantation to ensure survival. The severity of these disorders poses challenges for long-term clinical course monitoring, potentially resulting in an underestimation of the prevalence of bone involvement [29,30,31].

IL12p40 deficiency is the third most common deficiency reported in MSMD. Both IL12 and 23 are heterodimers having the IL12p40 subunit in common encoded by *IL12B*. Both IL12 and IL23 signaling are defective in patients with IL12p40 deficiency. Complete IL12p40 deficiency is a clinical phenocopy of IL12Rβ1 deficiency [20,32].

Unlike IFNγR1 deficiency, two forms of complete IFNγR2 deficiency have been reported with or without cell surface expression. The clinical presentation is similar to that of complete IFNγR1 deficiencies. In partial IFNγR2 deficiency, cell surface expression is weak, and response to IFNγ is impaired. The clinical phenotype is milder. Expression of IFNγR2 is very low in AD IFNγR2 deficiency. These deficiencies display incomplete penetrance [33,34].

Mutations in the *STAT1*, *JAK1*, *IRF8*, *SPPL2A*, *NEMO*, and *CYBB* genes are responsible for phagocytic defects leading to susceptibility to mycobacterial infections. JAK1 is a tyrosine kinase that induces phosphorylation and homodimerization of STAT1 upon binding of IFNγ to the receptor. Impaired response to IFNγ in phagocytes is seen in patients with JAK1 and STAT1 deficiencies. -Various forms of inherited STAT1 deficiency have been documented, with autosomal inheritance causing complete or partial STAT1 deficiency. Complete recessive STAT1 deficiencies are a result of the null mutations. The patient’s cells display unresponsiveness to IFN-γ and IFN-α/β. Consequently, these patients are susceptible to severe infections with mycobacteria and viruses. On the other hand, missense mutations lead to partial recessive STAT1 deficiency, resulting in an impaired but not abolished ability to respond to IFN-γ and IFN-α/β, leading to infections with intracellular bacteria and viruses.

Autosomal dominant STAT1 deficiency can result in loss-of-function (LOF) and gain-of-function (GOF) depending on underlined mutations. LOF mutations alter both IFNγ and IFN-α responses but have a negative dominant effect on the IFNγ response only. These patients typically develop mycobacterial infections without viral infections.

Conversely, mutations resulting in STAT1 gain of function predominantly occurring in the coiled-coil domain and DNA binding domain lead to increased phosphorylation and GAS-binding activity. Cells of affected patients exhibit an enhanced response to IFN-γ, IFN-α, and IL27. These patients commonly develop chronic mucocutaneous candidiasis (CMC) [35,36,37,38,39,40,41,42].

NEMO (NF-kappa-B essential modulator ) is a regulatory subunit of the nuclear factor-ĸB (NFĸB) inhibitor kinase complex. Impaired CD40-dependent IL12 production is responsible for the clinical phenotype of patients with NEMO deficiency [43,44]. TYK2 (Tyrosine kinase 2) is a Janus kinase that, upon stimulation with IL12 and 23, induces phosphorylation and dimerization of STAT4 and STAT3. Mutations in *TYK2* lead to susceptibility to mycobacterial and viral infections. Homozygosity for the allele of *TYK2 P1104A*, is a rare genetic etiology of MSMD that selectively disrupts IL23 responses. Patients with defects in RORC are susceptible to mycobacteriosis and candidiasis due to impaired IFNγ and IL17 immunity [8]. Disease-causing mutations in *CYBB* are known to cause chronic granulomatous disease. The hemizygous mutations Q231P and T178P in *CYBB* result in diminished NADPH oxidase activity, specifically in monocyte-derived macrophages. In contrast to individuals with Chronic Granulomatous Disease (CGD), neutrophils, monocytes, and monocyte-derived dendritic cells (MDCs) exhibit an unaffected respiratory burst. Since macrophage respiratory burst is essential in an immune response against mycobacteria, these variants have been included under MSMD [45]. A complete absence of monocytes and dendritic cells is seen in AR IRF8 deficiency, leading to susceptibility to mycobacterial and fungal infections. AD IRF8 deficiency leads to an absence of myeloid dendritic cells and susceptibility to mycobacterial infections. SPPL2A deficiency accumulates CD74 NTF in HLA class II+ myeloid and lymphoid cells. Accumulation of toxic fragments leads to the depletion of IL-12- and IL-23-producing CD1c+ conventional dendritic cells [46]. *IL12RB2 and IL23R* are the newly discovered but rare deficiencies that also underlie MSMD. Innate lymphoid cells (ILC1 and ILC2) exhibit a response to IL12, while ILC3, on the other hand, demonstrates responsiveness to IL23, resulting in the production of IFNγ. IFNγ production is compensated for in the absence of either of the cytokines [12]. Therefore, *IL12RB2* and *IL23R* defects have lower clinical penetrance than IL12Rβ1 deficiency. In T bet deficiency, patients suffered from BCGosis due to impaired IFNγ production by innate and innate-like lymphocytes [14]. ZNFX1 is essential for monocyte homeostasis, and deficiency leads to susceptibility to mycobacterial disease [15]. Mononuclear myeloid cells from patients with IRF deficiency have an impaired response to IFNγ, leading to susceptibility to mycobacteria [16]. The MAIT and Vδ2+ gδ T-lymphocyte subsets of the MCTS1-deficient patients generate small amounts of IFNγ in response to IL23 and BCG [17]. Impaired immunity due to the monogenic defects in genes involved in the IL12/23/ISG15-IFN-γ circuit results in clinical phenotypes of MSMD.

## 3. Infectious Spectrum of MSMD

**Figure 2:**
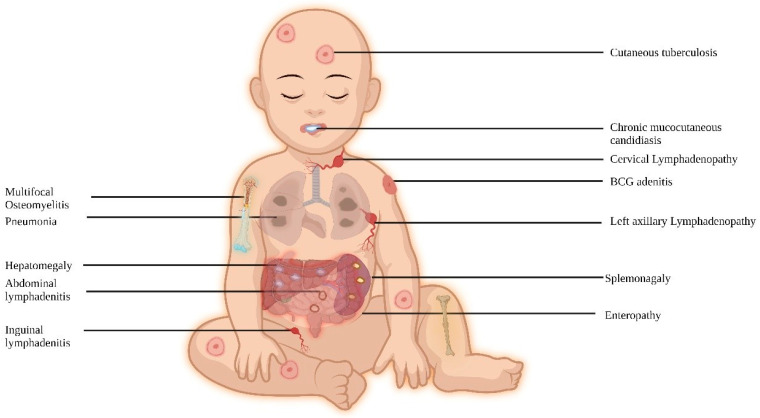
Clinical features of MSMD.

MSMD patients present with a diverse infection spectrum (Table 1). In regions where BCG is not administered at birth, NTM infections are the typical clinical manifestations, while in BCG-administering countries, complications related to BCG are commonly observed. These patients are also vulnerable to intracellular organisms, and the site of infection can range from localized to disseminated, impacting various organs (Figure 2).

Usually, BCG infection after vaccination is the most common infection in MSMD patients. BCG is a live-attenuated vaccine administered at birth in some countries. It is made from the subculture of *Mycobacterial* spp. i.e., *Mycobacterium bovis* (*M. bovis*). Currently, Brazilian (Moreau/Rio de Janeiro), Danish (Copenhagen–1331), Pasteur-1173, Japanese (Tokyo–172–1), Russian (Moscow–368), and Bulgarian (Sofia–SL222), Connaught-Toronto, Merieux, Chinese-Shanghai, and TICE strains are used for BCG vaccine production around the globe [47]. More than 170 countries give BCG vaccines as part of their national immunization programs. WHO recommends one dose to all neonates in countries with high TB burdens. High-risk groups such as children and healthcare workers with prolonged exposure are targeted in countries with low incidences (http://www.bcgatlas.org, accessed on 13 February 2024).

In the general population, BCG is proven to be safe, but adverse effects of vaccination can be seen in infants with underlying inborn errors of immunity (IEI) [48,49,50]. BCG-itis is a localized BCG infection observed as a local abscess, a severe ulcer at the injection site, or involvement of the regional ipsilateral ganglia with suppuration, fistula formation, or both. BCGosis is a life-threatening disseminated infection involving distal lymph nodes, skin, lungs, bone, liver, spleen, and the central nervous system (CNS). An adverse effect of the BCG vaccination is usually present within the first 6 months or beyond 12 months. Along with genetic factors, response to BCG vaccination is also dependent on the BCG strain used, storage of the vaccine, inappropriate dose, and injection technique.

A review of the literature revealed several reports on BCG complications in MSMD (Table 2). In MSMD, where countries give regular vaccinations, the proportion of patients manifesting with BCG complications varies between 35–100%. Adenitis varies between 8–40% versus severe BCGosis, which was 25–85% (Table 2).

This real-world data signifies the screening of MSMD patients in the event of BCG complications from BCG vaccination. Very often, the diagnosis is delayed in patients with BCG complications since they are not investigated. Very often, microbiological evidence is not always available, and a diagnosis is made solely based on clinical findings. This is due to a lack of awareness or diagnostic facilities. Early screening of these patients not only reduces the delay in diagnosis but also reduces morbidity and mortality. BCG vaccination can be delayed in patients with a strong family history of sibling death due to BCG complications and must be investigated.

However, it is essential to consider that BCG-related complications may also manifest in other IEIs, as indicated in Table 3, which depicts the prevalence of these IEIs among patients with BCG complications and needs to be evaluated. These alternative IEIs, including severe combined immunodeficiency (SCID), chronic granulomatous disease (CGD), X-linked hyper IgM syndrome (HIGM), signal transducer and activator of transcription 1 (STAT1) gain of function (GOF), hyper IgE syndrome (HIGE), and activated phosphoinositide 3-kinase δ syndrome (APDS), have been reported to exhibit BCG complications [51,52].

**Table 2:** Different countries with diagnosed cases of MSMD having BCG complications.

Study	Year	Origin	No. of MSMD Patients	% of Patients Presenting with BCG Complications	BCG Adenitis	BCGosis
1. Xia et al. [53]	2022	China	65	92%	29%	63%
2. Vicuña et al. [54]	2022	Mexico	22	63%	36%	27%
3. Mahdaviani et al. [55]	2022	Iran	24	100%	33%	67%
4. Prasad Taur [56]	2021	India	55	84%	-	84%
5. Cornelissen et al. [57]	2021	South Africa	22	64%	9%	55%
6. Elif Azarsiz [58]	2021	Turkey	12	50%	17%	33%
7. Radwan et al. [59]	2021	Mexico	11	36%	-	36%
8. Narmeen Galal [60]	2012	Egypt	9	78%	-	78%

Very little literature is available on MTB infections in MSMD. A large series of IL12Rβ1 patients reported only 6 patients with isolated *Mycobacterium tuberculosis* infections [67,68,69,70], and in this group, mycobacterial infections caused by BCG and EM are more common. In countries where BCG vaccination is mandatory, patients develop BCG disease, as a primary course of infection. Once diagnosed with BCG disease these patients do not acquire secondary mycobacterial infections. In TB-endemic countries, BCG disease may prevent subsequent EM disease but not MTB [20]. This is because M. tuberculosis has a close phylogenetic relationship with BCG and is more virulent than EM. Therefore, patients develop recurrent TB infections. The study from South Africa reported 22 patients with MSMD, and 64% of patients were positive for MTBC. Out of which, 55% of patients had recurrent episodes of TB involving the lungs, central nervous system, and gastrointestinal tract [57]. In a cohort of 25 patients diagnosed with IL12Rβ1 deficiency from India, 11 patients developed recurrent TB involving organs such as the lungs, abdomen, brain, skin, and kidney. IL12Rβ1, IFNγR1, STAT1, IL12p40, CYBB, and TYK2 P1104A deficiencies are found to be the commonest deficiencies associated with disseminated, extra-pulmonary, or recurrent TB [9,10,12].

The prevalence of NTM infections is high in countries where BCG vaccination is not administered. NTM mostly affects the lungs, but infections of the skin, soft tissue, and lymph nodes can also be seen [71]. In younger children, single-site lymphadenitis is the most common manifestation of NTM infection in low TB-endemic countries [72]. NTMs are defined as species of the Mycobacterium genus, excluding Mycobacterium tuberculosis (MTB complex) and Mycobacterium leprae. They are also called atypical mycobacteria or environmental mycobacteria since they habitat for food, soil, and water in the environment [73]. To date, 200 species of non-tuberculous mycobacteria have been identified [64]. They are further classified as rapid-growing or slow-growing mycobacteria, depending on how fast they can be grown in the laboratory. NTM diseases due to *Mycobacterium avium* and the Mycobacterium avium complex are commonly seen in patients with MSMD. These patients present with fever, night sweats, weight loss, abdominal pain, and diarrhea and may present with anemia, hepatosplenomegaly, and lymphadenopathy. Disseminated infection of the lung, spleen, multiple lymph nodes, and vertebrae due to *M. colombiense* is also reported [54]. *Mycobacterium chelonae* and *Mycobacterium fortuitum* are the second-most common infections reported in MSMD patients. *Mycobacterium genavense* and *Mycobacterium simiae* cause disseminated NTM disease in MSMD patients. Mycobacterium avium complex comprising *M. avium* and *M. intracellulare*, *Mycobacterium ulcerans*, and *Mycobacterium marinum* are the slow-growing mycobacteria (SGM). *Mycobacterium fortuitum*, *Mycobacterium abscessus*, and *Mycobacterium chelonae* are rapidly growing mycobacteria (RGM) that cause disseminated NTM infections in MSMD patients. In MSMD patients, 45% of cases with RGM have been reported, which includes *Mycobacterium abscessus* (32%) and *Mycobacterium fortuitum* (12%) [71]. High morbidity and up to 52% mortality due to NTM infections have been reported in MSMD patients [53].

About half of MSMD patients also develop non-typhoidal *Salmonella* infections [19,32]. Infections can cause non-typhoidal salmonellosis or typhoid-like disease [74]. Invasive salmonellosis is the second most common infection seen in 38% of IL12Rβ1-deficient patients and 25% of IL12p40-deficient patients [19,75,76,77]. Patients often present with multiple episodes of infections. They present with extraintestinal non-typhoidal salmonellosis alone or both mycobacteriosis and salmonellosis [19]. Leukocytoclastic vasculitis is seen in some cases [20,78]. Extraintestinal manifestations such as multiple lymph adenitis, arthritis, Henoch–Schönein purpura, sepsis, and osteomyelitis may be seen in patients with IL12Rβ1 deficiency [10,79]. Salmonella infections remain undiagnosed in most of the IL12Rβ1 patients due to the accompanying mycobacterial infections and antibiotics used for treatment [20]. IFNγR1 deficiency is associated with only 5% of salmonella infections [25].

## 4. Challenges Associated with a Diagnosis of MSMD

### 4.1. Microbiological Diagnosis

Early identification of mycobacterial infections in MSMD patients is essential, as treatment strategy depends on the type and sensitivity pattern of the mycobacteria isolated. However, differential diagnosis of TB and NTM is a significant challenge in TB-endemic countries. In TB-endemic countries, the incidence of NTM infection is often underestimated due to a lack of awareness of the NTM disease and poor or inadequate diagnostic methods for its identification. The clinical symptoms of NTM and MTB infections are similar. Therefore, NTM infections can be misdiagnosed as MTB infections. The decision to treat is also made without confirmation of mycobacterial species. This may give rise to drug resistance in a few patients. Despite the presence of several assays, diagnosing tuberculosis remains a challenging endeavor.

The conventional method for TB diagnosis can be categorized into direct and indirect methods. Direct methods could be based on phenotype modalities such as microscopy using acid-fast or florescent stains, culture on solid or liquid media (automated MGIT 960 being recommended), or molecular modalities such as CBNAAT (GeneXpert MTB/RIF, RIF ultra), True NAAT, and other molecular platforms. The molecular platform usually targets only MTBC and can miss NTM even when present unless it specifically probes for them. Among the current available phenotypic tests, culture is considered the gold standard. It can grow and differentiate NTM from MTBC. Culture has a long turnaround time considering the slow-growing nature of MTBC. Deep genome sequencing technique-based platforms are under evaluation. These can detect up to 100 *Mycobacterial* spp. directly from the specimens.

Indirect methods hinge on the host immune response to Mycobacterium tuberculosis complex (MTBC) antigens. Among the earliest techniques is the Mantoux skin test, which relies on a delayed-type hypersensitivity (DTH) reaction to purified protein derivatives (PPD). The QuantiFERON-TB Gold Plus assay identifies the release of interferon-gamma (IFNγ) after exposure to MTBC-specific antigens. However, neither of these methods is generally recommended for diagnosing active tuberculosis. Caution is advised in interpreting QuantiFERON-TB Gold Plus results, as IFNγ production may be defective in many genes associated with MSMD, rendering it unreliable for ruling out active TB infections in MSMD patients [80]. Also, the presence of anti-IFN-gamma autoantibodies can lead to an indeterminate result in Interferon-Gamma Release Assays (IGRA), as these antibodies hinder the detection of IFN-gamma [81].

The incubation temperature for certain NTMs is lower (28 ± 2 °C for rapidly growing *Mycobacterial* spp.), whereas others require a hyper-optimum temperature, such as *M. xenopi* (42–45 °C). Differentiating mycobacteria growing in culture-based systems requires a battery of phenotypic biochemical tests, which are both time-consuming and labor-intensive. Simple, rapid immune chromatography-based tests that detect MPT64 antigen and MTBC are also available. In recent years, MALDI TOF MS-based systems have been available for rapid and accurate identification of the most clinically relevant mycobacteria. Molecular-based platforms have the advantage of both accuracy and rapid turnaround time compared to phenotypic culture-based tests.

With the increasing incidence of DR (drug resistance) TB, drug susceptibility testing by culture or detection of mutations contributing to the DR has become imperative, and commercial systems are available for the same. The utility of WGS in detecting the mutation is currently under consideration. The choice of method/test would depend on the present probability of an MTBC infection in most pulmonary TB, or sometimes associated with extrapulmonary TB. As a matter of practice, liquid culture followed by molecular methods can detect and differentiate most *Mycobacterial* spp., including *M. bovis* BCG strains.

### 4.2. Immunological Evaluation

Children and adults with recurrent and disseminated mycobacterial infections, Salmonella infections, infections with other intracellular organisms, and a family history of invasive and recurrent mycobacterial infections are tested for MSMD. Before performing specific MSMD tests, acquired and inherited immunodeficiencies that confer susceptibility to various diseases, including mycobacteria such as SCID and CGD, and predominantly antibody deficiencies must be ruled out by doing lymphocyte subset analysis (LSS), the nitroblue tetrazolium test (NBT), the dihydrorhodamine assay (DHR), and serum immunoglobulin levels. Cytokine production is an important tool in evaluating an inborn error of the IFNγ underlying MSMD. This assay helps to distinguish between IFNγ production and response defects. Complete deficiencies such as IFNGR1, IFNGR2, IL12Rβ1, or IL12p40 can be detected using this assay, but CYBB or AD IRF8 deficiency shows normal IFNγ production. Also, partial defects cannot be identified clearly with this approach. Detection of baseline IFNγ in plasma or serum using ELISA is a simple technique that can help in identifying complete defects. Patients with partial defects have detectable levels of IFNγ. In acute mycobacterial disease, levels vary as compared to the convalescent phase. So IFNγ level measurement is performed after the resolution of acute infection. Flow cytometric determination of receptors can be an easy and fast tool to detect deficiencies along with signaling assays as non-functional receptors may express on the surface. Flow cytometric detection of IL12Rβ1 expression requires stimulation with PHA antibodies available for surface expression of IFNγR2, which is not optimal [9].

### 4.3. Genetic Diagnosis

**Figure 3:**
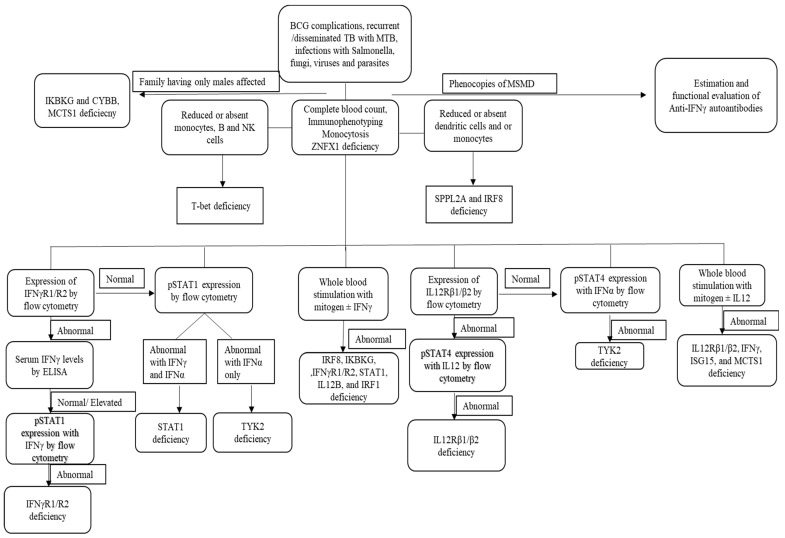
Overview of functional assays in patients with MSMD.

The diagnosis of MSMD is finally confirmed by the identification of pathogenic variants, either by Sanger sequencing or next-generation sequencing. A targeted panel with known genes can also be an option. However, half of the patients exhibiting clinical signs indicative of MSMD do not display mutations in known MSMD-causing genes [9]. Next-generation sequencing will help MSMD patients without a genetic diagnosis. NGS will be less time-consuming and may cost less [82]. NGS will also lead to the discovery of new genetic etiologies and the identification of mutations missed by Sanger sequencing, such as copy number variation (CNV) [83]. Identification of novel mutations requires further specific tests for functional validation of genes to confirm the underlying defects (Figure 3).

## 5. Management of Patients with MSMD

The management of patients with MSMD depends on the underlying genetic defect. It primarily involves the control of mycobacterial and other infections and hematopoietic stem cell transplantation. Interferon-γ therapy plays an important role in selected cases. There are some case reports on the use of gene therapy for the correction of the underlying defect.

Management of BCG-related complications remains a challenge since there are no evidence-based guidelines for the selection of appropriate antimicrobial regimens. The treatment decisions are made on a case-by-case basis in the background of tuberculosis management guidelines by the WHO and national TB control bodies. Since BCG is resistant to pyrazinamide (PZA), it is not part of the treatment [84]. Some studies have reported low-level resistance to INH against the Danish 1331 strain. It is recommended that ATT be initiated without waiting for the genetic confirmation of the disease according to the sensitivity pattern of the organism [85].

All patients suspected of having a BCG disease should undergo an X-ray of the chest to rule out pulmonary TB. Ultrasound or CT scans of the chest and abdomen may be performed where a disseminated disease is suspected. Fine needle aspiration cytology of the lymph node by non-dependent aspiration with Z-technique for manipulating overlying skin by an appropriately trained operator, and the aspirate should be sent for part MTB/RIF test, microscopy, and culture for Mtb with drug susceptibility testing, and a cytology smear. In cases where the FNAC is inconclusive, an excision biopsy should be performed, and the specimen must undergo an Xpert MTB/RIF test, microscopy, and culture for MTB with drug susceptibility testing and histopathological evaluation.

In patients with BCGiosis, a prolonged course of modified ATT is required depending on the organ involvement, which may range from 9–12 months. In some cases, the ATT may need to be given for a prolonged period of 18 months. Though there is no definite guideline, patients usually respond to second-line ATT medications, so early initiation of second-line ATT, including fluoroquinolone and aminoglycosides, is beneficial. Depending on the degree of dissemination, second-line anti-TB drugs, including Linezolid, a quinolone (e.g., ciprofloxacin, levofloxacin), an aminoglycoside (e.g., amikacin, streptomycin), and clarithromycin, are used. Once the infection is optimally controlled, a prophylactic regimen with two drugs (usually INH plus RFP) should be continued until the patient is ready to be taken up for transplantation. At the end of therapy, a biannual follow-up may be required for at least two years, depending on the etiology. Treatment failure needs to be considered if there is no response after five months of ATT.

The conventional treatment guidelines for drug-sensitive (DS) TB involve a six-month regimen of a two-month intensive phase of INH/RFP/PZA/EMB followed by a four-month INH/RFP/EMB course. Recently, a shorter course of four months was recommended by the WHO for children > 3 years old in the background of the SHINE trial in patients with a non-severe form of TB. EMB should be a part of the intensive phase in countries with a high prevalence of HIV and INH resistance. Even for patients with HIV infection, depending on the degree of immunosuppression, a four-month regimen may be used with careful monitoring. Because the majority of patients with BCG adenitis present before three months of age, these patients should be promptly treated with the 6-month treatment regimen without PZA. Some patients with residual lymphadenopathy of less than 1 year at the end of treatment are labeled as partial responders, and there is uncertainty about whether continuation of ATT in these patients is beneficial. An additional three months of INH/RFP/EMB, followed by a biopsy sent for histology and TB culture in patients who fail to respond to that, is recommended.

In patients with a minimal or temporary response to ATT, non-tuberculous mycobacterial infections should be considered. Newer ATT drugs such as Bedaquiline have in vitro activity against NTMs such as *M. avium* complex, *M. abscessus*, *M. ulcerans*, and *M. smegmatis* [35]. In gamma interferon knockout (GKO) mice and SCID mice, bedaquiline alone and in combination with clofazimine reduced bacterial burden in the lung, spleen, and liver [86]. Once the patient stabilizes, HSCT as the only curative treatment option may be considered.

In patients with partial IFN-γ receptor deficiency, after the resolution of the acute symptom, lifelong azithromycin prophylaxis is successful in preventing recurrences. The IFN-γ receptor deficiencies are generally severe and require aggressive long-term antimicrobial therapy. The IL-12R pathway defects generally tend to be milder, and the need for prophylaxis is unclear [87]. Though in our experience, some IL12RB1-deficient patients can have recurrent infections.

IFN-γ therapy restores macrophage function and provides better control of BCGiosis. In patients with impaired or intact IFN-γ responses, IFN-γ therapy is effective [84]. In patients with AR-complete IFNγR1 or IFNγR2 deficiency, no response to IFN-γ therapy is seen. In patients with partial IFNGR1 and IFNGR2 deficiency, IFNγ is usually administered by a subcutaneous route three times a week with a dose of 30–50 mg/m^2^. If a poor response is observed, the dose is gradually increased at monthly intervals until a response is observed, with the maximum dose being 400 mg/m^2^.

Some patients with IL12–23 component disease are at increased risk of salmonellosis [88]. These patients are treated with appropriate antibiotics. A combination of ceftriaxone and ciprofloxacin is reported to have a good response. In patients with recurrent salmonellosis, prophylaxis with trimethoprim-sulfamethoxazole (cotrimoxazole) is required [89].

## 6. Hematopoietic Stem Cell Transplantation

Hematopoietic stem cell transplantation (HSCT) is the only curative option for patients with AR complete *IFNGR1* and *IFNGR2* deficiency, though the overall prognosis was reported to be poor. The use of different therapeutic and prophylactic protocols, including anti-IFN-γ, may result in better outcomes in these cases. It has also been performed for patients with *IL12RB1*, *NEMO*, and *IRF8* deficiencies. Donor selection has a significant impact on outcomes with fully matched related or unrelated donors having better outcomes as compared to haploidentical donors [90]. High failure rates were reported in patients with IFNG receptor deficiencies because of the recurrence of infections and high rates of graft versus host disease because of high serum IFN-γ levels, which inhibit stem cell proliferation and hematopoiesis [91].

## 7. Gene Therapy

Gene therapy has been documented to be helpful in select IEIs, including ADA SCID, X-linked SCID, Wiskott-Aldrich syndrome, X-linked CGD, and leukocyte adhesion deficiency type 1 [92]. The self-inactivating lentiviral vector, which targets primitive hematopoetic stem cells, has led to a better safety profile and efficacy [93]. A recent study described a lenti-viral-based gene therapy approach to correct cellular phenotypes in AR patients with complete IFNγR1 deficiency [94]. A mouse model with complete IFNGR1 deficiency was transplanted using lentiviral-based gene therapy [95]. The transplant protected mice from severe BCG disease. This novel therapeutic approach will prove helpful in patients with IFNγR1 deficiency, especially with the limited success of HSCT in these cases.

## 8. Conclusions

Multiple genes are involved in MSMD with clinical overlap, which delays the diagnosis and management of patients with MSMD. Appropriate microbiological, immunological, and genetic approaches can help in the rapid diagnosis of MSMD. A newer gene therapy approach using lentiviral vectors to correct the AR-complete IFNγR1 deficiency can be the future management option in MSMD.

## Figures and Tables

**Table 1:** The spectrum of infections seen in MSMD.

	Clinical Manifestations	Mycobacterial Infections	Bacterial Infections	Viral Infections	Fungal Infections	Parasitic Infections	References
AR complete IFN-γR1 deficiency	Skin lesions, Pneumonia, B-cell lymphoma, pineal germinoma, and Kaposi sarcoma	MAC, disseminated *M. kansasii*, *M. chelonae*, *M. fortuitum*, *M. mageritense*, *M. peregrinum*, *M. smegmatis*, *M. scrofulaceum*, *M. tuberculosis*, *M. tuberculosis*	*Listeria monocytogenes*, *Streptococcus*	Cytomegalovirus (CMV), varicella-zoster virus (VZV), human herpes virus-8 (HHV8), piscine ortho-reovirus subtype 3 (PRV-3), respiratory syncytial virus (RSV)), parainfluenza virus (PIV)			[9,25,26,27,28]
AR partial IFN-γR1 deficiency	Osteomyelitis	BCG, *M. avium*, *M. avium* complex,* M. abcessus*, *M. szulgai*, *M. tuberculosis*	*Salmonella* sp., *Klebsiella pneumoniae*, *Haemophilus influenzae*, *Legionella* sp., *Mycoplasma pneumoniae*, *Shigella sonnei*	VZV, molluscum contagiosum, RSV		*Toxoplasma gondii*, *Cryptosporidium* sp.	[9]
AD partial IFN-γR1 deficiency	Osteomyelitis	BCG, *M. abcessus*, *M. avium* complex, *M. asiaticum*, *M. bohemicum*, *M. chelonei*, *M. gordonae*, *M. kansasii*, *M. scrofulaceum*, *M. bovis*, *M. tuberculosis*, *Coccidiodes* sp.	*Salmonella* sp.	VZV	*Histoplasma capsulatum*, *Cocciodiodes* spp.		[9,25]
AR complete IFN-γR2 defect	cutaneous squamous cell carcinoma	BCG, *M. abscessus*, *M. avium*, *M. fortuitum*, *M. porcium*, and *M. simiae*	*Salmonella* sp.	CMV			[9,33]
AR partial IFN-γR2 defect	Osteomylitis	BCG, *M. abscessus*, *M. bovis*, *M. elephantis*, *M. fortuitum*, and *M. simiae*					[9]
AD partial IFN-γR2 defect	Osteomylitis, lymphoreticular, pulmonary, gastrointestinal, central nervous system	BCG disease, *M. bovis*					[9]
IFN-γ deficiency	disseminated BCGosis						[13]
IL12Rβ1 deficiency	Chronic mucocutaneous candidiasis, leukocytoclastic vasculitis, esophageal carcinoma, Inflammatory bowel disease (IBD)	BCG, *M. avium*, *M. avium* intracellulare complex, *M. chelonae*, *M. fortuitum*, *M. fortuitum-chelonae* complex, *M. genevense*, *M. gordonae*, *M. tilburgii*, *M. triplex*, *M. simiae*	*S. enteritidis*, *S. typhimurium*, *S. dublin*, *S. hadar*, *S. typhi O* and typhi H, S. group B and D, *S. portland*, *S. paratyphi*, *Klebsiella pneumoniae*, *Nocardia*		*Candida* sp., *Cryptococcus neoformans*, *Coccidiodes* sp., *Paraccocidiodes brasiliensis*, and *Histoplasma* sp.	*Toxoplasma* and *Leishmania*	[8,21,22,23,19]
gp91phox deficiency Q231P and T178P	disseminated BCGosis and regional BCGitis	*M. tuberculosis*		*Herpes zoster*			[9,45]
NEMO deficiency	*Cervical abscess*	disseminated mycobacterial diseases (Mycobacterium avium complex is the most common); recurrent BCG infection	*Haemophilus influenzae* type b, *Enterobacter* sp., and S*almonella* sp.				[9]
IRF8 deficiency	disseminated BCGosis, calcifications in the brain parenchyma, oral candidiasis	BCG		*Viral infections*	*Candida* spp.		[9]
STAT1 deficiency	Osteomyelitis	BCG, *M. avium*, *M. szulgai*, *M. tuberculosis*	Salmonella	EBV, CMV and VZV			[9,36,38,41]
ISG15 deficiency	Intracranial calcifications, recurrent inflammatory necrotizing ulcerative skin lesions, Pulmonary involvement, epileptic seizures	BCG	*E. coli*, and methicillin-resistant *Staphylococcus aureus*		*Aspergillus* sp.		[9]
IL-12B deficiency	BCG disease	BCG, *M. tuberculosis*	*Salmonella*, *Nocardia* sp., and *Klebsiella* sp.		*Candida* sp.		[9,20,32]
IL-23 receptor deficiency	BCGadenitis, BCGosis	BCG disease					[12]
IL-12Rβ2 deficiency	pulmonary TB	BCG disease					[12]
TYK2 deficiency	BCG disease, atopy	BCG and M. tuberculosis	Staphyalococcus sp., Other intracellular bacteria	Herpes virus family	*Candida* spp.		[8]
SPPL2A deficiency	BCG lymphadenitis, spastic paraplegia	BCG					[8,12,46]
RORγ deficiency	CMC, with onychomycosis, disseminated BCG disease, Hypoplasia of the thymus and impaired lymphoid development, pneumonia	BCG, Mycobacterium tuberculosis					[8]
JAK1 deficiency	recurrent sinopulmonary infections, global developmental delay, bladder carcinoma	mycobacterial infection		Viral infections			[8]
T-bet deficiency	BCGosis in early infancy, reactive airway disease	BCG					[14]
USP18 deficiency	Intracranial calcifications	BCG					[18]
ZNFX1 deficiency	BCGosis, recurrent respiratory infections, pneumonia, and interstitial lung disease	BCG		CMV			[15]
IRF1 deficiency	BCG adenitis, BCGosis	BCG, *M. avium* intracellulare		Varicella zoster virus	Histoplasma		[16]
MCST1 deficiency	BCGosis, osteomyelitis	BCG					[17]

**Table 3:** BCG complications associated with IEI.

Study	Year	Origin	Total No. of IEI Patients with BCG Complications	No. of SCID Patients	No. of CGD Patients	No. of MSMD Patients	No. of Other IEI Patients
1. Bernatowska et al. [61]	2022	Poland	180	72	63	6	39
2. Paula, T. Lyra [62]	2022	Brazil	9	2	4	3	-
3. Reetika Yadav [63]	2020	India	52	13	15	19	5
4. Rina yue Ling Ong [64]	2020	Singapore	10	4	-	3	3
5. M. Sohani [65]	2020	Iran	137	75	15	47	-
6. Lie et al. [66]	2010	China	12	3	7	2	-
7. Santos et al. [66]	2010	Portugal	3	1	-	2	-
8. Lee et al. [66]	2009	Taiwan	18	12	2	3	1
9. Toida and Nakata [66]	2007	Japan	19	4	5	4	6
10. Bustamante [66]	2007	France	3	-	-	3	-
